# P wave indices, heart rate variability and anthropometry in a healthy South Asian population

**DOI:** 10.1371/journal.pone.0220662

**Published:** 2019-08-23

**Authors:** James O’Neill, Katrina Bounford, Alice Anstey, Jesvita D’Silva, Lisa Clark, Sven Plein, Muzahir H. Tayebjee

**Affiliations:** 1 Department of Cardiology, Leeds Teaching Hospitals NHS Trust, Leeds General Infirmary, Leeds, United Kingdom; 2 Multidisciplinary Cardiovascular Research Centre (MCRC) and Leeds Institute of Cardiovascular and Metabolic Medicine, University of Leeds, Leeds, United Kingdom; Indiana University, UNITED STATES

## Abstract

**Background:**

South Asians have a low prevalence of atrial fibrillation (AF) in comparison with White Europeans despite a higher burden of hypertension, diabetes mellitus and coronary artery disease. The reason for this disparity is unclear but may relate to electrophysiological or structural differences within the atria or variations in autonomic function. We aimed to assess these areas using a range of non-invasive cardiac investigations.

**Methods:**

A prospective cohort study was performed on 200 South Asian and 200 Caucasian healthy volunteers aged 18–40 years. All subjects underwent electrocardiography (ECG), echocardiography and anthropometric measurements. Eighty subjects in each cohort underwent 24 hour ambulatory ECG and fifty subjects in each cohort underwent exercise testing.

**Results:**

Compared with White Europeans, South Asians were of a smaller height with lower lean body mass and smaller left atrial size. They had reduced P wave dispersion and P wave terminal force in lead V_1_. South Asians had a lower burden of supraventricular ectopy. They had a higher mean heart rate and South Asian males had lower heart rate variability, suggestive of sympathetic predominance. Exercise capacity was lower in South Asians.

**Conclusions:**

South Asians have differences in left atrial size, P wave indices, burden of supraventricular ectopy, heart rate, heart rate variability and anthropometric measurements. These differences may relate to variations in atrial morphology, atrial electrophysiology and autonomic function and might help to explain why South Asians are less susceptible to developing AF.

## Introduction

Atrial fibrillation (AF) is the most common sustained cardiac arrhythmia[1] with an estimated prevalence of around 3% in adults aged 20 years or older[2]. Risk factors for the development of the arrhythmia include hypertension, diabetes mellitus and coronary artery disease[3]. These conditions are particularly prevalent amongst the South Asian population[4,5], a diverse ethnic group which makes up around a fifth of the world’s population, is the largest minority group in the United Kingdom[6] and represents one of the fastest growing immigrant populations in North America[7]. Despite a higher prevalence of cardiovascular risk factors, South Asians have been consistently shown to have a lower burden of AF compared with White Europeans and the reason for this disparity remains unclear[4,8].

The mechanisms underlying the development of AF are complex. The initiation of AF requires the formation of an ectopic focus which then acts as a trigger for the arrhythmia[9]. A combination of atrial enlargement and myocardial fibrosis facilitates the maintenance of AF through the development of multiple re-entrant circuits[9]. Alterations in autonomic tone are also recognised as playing an important role in the genesis of AF through the creation of ectopic foci and the effects on atrial refractoriness[10].

Non-invasive cardiac investigations can indirectly assess many of these mechanisms without the inherent risks of invasive testing. P wave indices measured on a 12 lead electrocardiogram (ECG) are a marker of atrial conduction, ambulatory ECGs can determine the burden of supraventricular ectopy (SVE) and heart rate variability (HRV), echocardiography can be used to measure atrial size and exercise testing can assess exercise capacity and heart rate recovery.

West Yorkshire, England, has a population of over two million,11.9% of whom are of South Asian descent[6]. It is therefore an ideal setting to study why South Asians have less AF. We hypothesized that South Asians would have differences in atrial electrophysiology and autonomic function compared with White Europeans and aimed to assess this by performing a range of non-invasive cardiac investigations. Healthy volunteers were studied to determine to effects of ethnicity on these measurements without potential bias from co-existent cardiovascular conditions.

## Methods

### Study population

A single centre prospective cohort study on healthy South Asian and Caucasian volunteers aged 18 to 40 years was performed between 14^th^ February 2017 and 14^th^ December 2017. Subjects were recruited from Leeds Teaching Hospitals NHS Trust, the University of Leeds and local community centres using poster and email advertisements. They were excluded if there was a history of cardiovascular disease, hypertension, diabetes mellitus or dyslipidaemia or if they had evidence of structural heart disease on initial screening. Adults over the age of 40 were not recruited in order to minimise the risk of subjects having undiagnosed cardiovascular disease. Ethnicity was self-reported and fell into two categories: South Asian (defined as Indian, Pakistani, Bangladeshi, Sri Lankan or Nepalese) and Caucasian (defined as White British or White European). All subjects lived in West Yorkshire and all were born in the United Kingdom except for four South Asians who were born in the Indian sub-continent and two White Europeans who were born in Europe.

After obtaining written informed consent, all subjects underwent anthropometric and blood pressure measurements, transthoracic echocardiography and 12-lead ECG. Based on our power calculations, a cohort of 160 volunteers underwent a 24-hour ambulatory ECG and 100 volunteers underwent exercise testing.

The study was approved by the London—Surrey Borders Ethics Committee (REC reference 16/LO/2220).

### Anthropometric measurements

All measurements were performed by a single investigator in order to reduce variability. Height was measured to the nearest millimetre using a stadiometer (SECA, model 799, Hamburg, Germany) with participants standing without shoes. Weight was measured to the nearest 100 grams using a digital scale (SECA, model 799, Hamburg, Germany) with participants in light clothing.

Waist and hip circumference were calculated to the nearest centimetre with an anthropometric tape (Gulick II tape measure, Country Technology, Wisconsin, USA) using previously published standard techniques[11]. Body mass index (BMI) and body surface area (BSA) were calculated by dividing weight by the square of the height and using the Mosteller method[12] respectively.

Body fat content was assessed using the skinfold thickness technique[13]. Bicep, tricep, subscapular and suprailiac skinfolds were measured in triplicate using a Harpenden skinfold caliper (HSK-BI, Burgess Hill, United Kingdom). Skinfold measurements were converted into body fat using the Durnin-Womersley method[14].

### Blood pressure measurement

Blood pressure was measured in duplicate after subjects had rested for 10 minutes using a semiautomatic validated device (model HEM-59, Omron Healthcare, Netherlands). Measurements were taken using an appropriate sized cuff on the right arm with the subject in a seated position.

### Transthoracic echocardiogram

A focussed transthoracic echocardiogram (Vivid S6 and Vscan, GE Healthcare, Milwaukee, USA) was performed on all volunteers to screen for evidence of significant (moderate or severe) valvular heart disease or left ventricular dysfunction. Measurements of left atrial diameter in the parasternal long-axis view were recorded.

### Electrocardiogram

Standard 12-lead ECGs were acquired digitally (Norav PC-ECG 1200, Norav Medical Ltd, Israel) at a calibration of 10mm/mV and a speed of 25mm/s. ECG data was analysed digitally (Resting PC-ECG Application version 5.514, Norav Medical Ltd, Israel) by a single operator who was blinded to the study population. Heart rate, baseline ECG intervals and P wave indices which have been shown to be associated with the development of AF[15–18] were recorded. Maximum, minimum and mean P wave duration (the onset and offset points of the P wave were defined as the intersection point of upward or downward deflection in relation to the isoelectric line) and P wave amplitude were measured in all 12 leads. P wave dispersion (derived from the difference between maximum and minimum P wave duration) and P wave terminal force in lead V_1_ (PWTF-V_1_, defined as the product of the duration and amplitude of the negative terminal portion of the P wave) were also calculated. Measurements were made manually using electronic calipers at a calibration of 40mm/mV and a sweep speed of 200mm/s.

### 24 hour electrocardiogram

Ambulatory ECG monitoring (Lifecard CF Holter, Spacelabs Healthcare, USA) was performed over a 24 hour period. Subjects were encouraged to continue their usual daily activities, including exercise. Recordings were digitally sampled and analysed (Pathfinder SL, Spacelabs Healthcare, USA) by two operators who were blinded to the study population. The burden of SVE or arrhythmia was recorded. HRV was measured after the manual removal of artefact and ectopy. Ambulatory ECGs with less than 18 hours of recording or with less than 90% of the recording suitable for analysis were excluded to avoid confounding effects of circadian variations in HRV.

Time-domain and frequency-domain measures of HRV were analysed according to previously published guidelines[19]. Time-domain measurements included the standard deviation of all normal RR intervals (SDNN), the standard deviation of the average normal RR intervals in all 5 minute segments of the entire recording (SDANN), the mean of the standard deviations of all normal RR intervals for each 5 minute segment of the entire recording (SDNN index), the square root of the mean of the sum of the squares of differences between adjacent normal RR intervals (rMSSD) and the integral of the total number of all normal RR intervals divided by the height of the histogram of normal RR intervals (HRV triangular index). Spectral analysis was performed using the fast Fourier transform method. Three frequency bands were calculated: very low frequency power (VLF, 0.017–0.049 Hz), low frequency power (LF, 0.05–0.15 Hz) and high frequency power (HF, 0.15–0.35 Hz).

### Exercise test

Treadmill stress testing was performed according to a standard Bruce protocol[20]. Once maximal workload was reached, subjects entered an active recovery phase with the treadmill at a speed of 1.5 miles/hour and a gradient of 2% before a passive recovery phase of four minutes. Heart rate recovery, defined as the difference in heart rate between peak exercise and after one minute of active recovery was recorded.

### Statistical analysis

Our power calculations were based upon the results of previous studies which had demonstrated a standard deviation (SD) of 12ms for P wave duration, 8ms for P wave dispersion, 33mV for P wave amplitude, 1.8mm•s for PWTF-V_1_, 0.5ms for SDNN and 9 beats-per-minute for heart rate recovery[21–23]. We calculated that a sample size of 400 subjects would have 80% power to detect a difference of 5ms for P wave duration, 2ms for P wave dispersion, 11mV for P wave amplitude and 0.6mm•s for PWTF-V_1_ at a 5% significance level. We calculated that a cohort of 160 subjects for ambulatory ECGs and 100 subjects for exercise tests would have 80%power to detect a difference of 0.1ms for SDNN and 6 beats-per-minute for heart rate recovery at a 5% significance level.

Statistical analysis was performed using SPSS (IBM SPSS Statistics Version 22.0, IBM Corporation, Armonk, New York). Normality of data was tested using a Shapiro-Wilk test. Continuous variables were expressed as mean ± SD if normally distributed or median (interquartile range [IQR]) if non-normally distributed. Student t test or Mann Whitney U test were used to compare continuous variables depending on normality. Categorical variables were expressed as percentages and compared using Pearson’s chi-square test. *P* values of less than 0.05 were considered statistically significant.

## Results

### Study cohort

A total of 200 South Asians and 200 White Europeans were recruited. All subjects underwent ECG, echocardiography and anthropometric measurements. Eighty individuals from each cohort underwent 24 hour ambulatory ECG and fifty individuals from each group underwent exercise testing (Table 1).

**Table 1:** Baseline characteristics.

	Male			Female		
	Caucasian	South Asian	p-value	Caucasian	South Asian	p-value
Subjects	100	100		100	100	
Age, years	28.0 (7)	28.0 (13)	0.757	23.5 (8)	22.0 (8)	0.687
Alcohol consumption, units/week	10.0 (16)	0.0 (2)	**<0.001**	5.0 (10)	0.0 (0)	**<0.001**
BP- systolic, mmHg	128.3±11.8	129.0±11.6	0.674	122.4±10.0	120.5±12.3	0.313
BP- diastolic, mmHg	77.7±8.5	78.1±9.8	0.781	78.8±8.8	79.2±9.7	0.720
Height, cm	180.5±6.3	173.8±6.8	**<0.001**	165.6±6.2	161.3±5.8	**<0.001**
Weight, kg	81.3 (19.4)	75.0 (16.8)	**<0.001**	62.6 (11.8)	59.5 (13.7)	**0.047**
Body mass index, kg/m^2^	24.6 (4.6)	24.2 (4.4)	0.226	22.9 (4.5)	22.9 (4.7)	0.546
Body surface area, m^2^	2.0 (0.3)	1.9 (0.3)	**<0.001**	1.7 (0.2)	1.6 (0.2)	**0.006**
Waist circumference, cm	82.0 (17)	83.0 (13)	0.658	71.0 (11)	74.0 (16)	0.059
Hip circumference, cm	99.0 (12)	96.0 (10)	**<0.001**	95.0 (10)	94.0 (12)	0.769
Waist:hip ratio	0.83 (0.1)	0.85 (0.1)	**0.023**	0.75 (0.1)	0.79 (0.1)	**<0.001**
Biceps SFT, mm	5.4 (3.3)	5.6 (4.1)	0.784	8.2 (4.1)	9.2 (4.8)	**0.048**
Triceps SFT, mm	11.9 (6.7)	10.6 (7.7)	0.282	18.7 (7.7)	17.7 (8.5)	0.476
Subscapular SFT, mm	13.1 (8.4)	15.9 (8.5)	**0.003**	14.1 (6.6)	18.8 (8.8)	**<0.001**
Suprailiac SFT, mm	23.6 (13.8)	27.0 (14.6)	**0.027**	20.1 (11.0)	26.3 (9.6)	**<0.001**
Body fat, %	21.9±5.4	22.6±5.4	0.356	29.2±4.7	31.4±5.0	**0.001**
Fat mass, kg	17.5 (8.9)	16.4 (7.9)	0.301	17.6 (6.3)	18.1 (8.2)	0.538
Lean body mass, kg	63.4 (12.1)	57.8 (10.2)	**<0.001**	45.4 (7.0)	41.5 (7.3)	**<0.001**
LA diameter, cm	3.3 (0.6)	3.0 (0.4)	**<0.001**	2.8 (0.4)	2.6 (0.5)	**0.001**
LA diameter/BSA, cm/m^2^	1.64 (0.1)	1.58 (0.1)	**<0.001**	1.64 (0.0)	1.58 (0.1)	**0.008**

Values displayed are mean ± SD or median (interquartile range).

Abbreviations: BP, blood pressure; SFT, skinfold thickness; LA, left atrial, BSA, body surface area.

### Anthropometric measurements

South Asians were of a significantly smaller height, lower body weight, reduced BSA and lower lean body mass in comparison with White Europeans (Table 1). Waist:hip ratio was higher in South Asians but there was no difference in BMI. Importantly, South Asians had evidence of a smaller left atrium, even after correction for BSA.

### 12-lead ECG and P wave indices

South Asians had a narrower QRS complex in comparison to White Europeans and South Asian females also had a shorter QTc interval (Table 2). There was no difference in the PR interval or cardiac axis.

**Table 2:** Reference ranges for South Asian and Caucasian ECG and P-wave indices.

	Male			Female		
	Caucasian	South Asian	p-value	Caucasian	South Asian	p-value
Heart rate, beats/minute	63.0 (16)	67.0 (17)	**0.004**	72.0 (17)	72.0 (15)	0.960
PR, ms	154.0 (28)	150.0 (29)	0.134	144.0 (22)	144.0 (32)	0.889
QRS, ms	84.0 (10)	78.0 (6)	**<0.001**	76.0 (8)	74.0 (6)	**0.002**
QTc, ms	387.0 (30)	382.0 (24)	0.173	400.0 (29)	395.0 (27)	**0.038**
P axis	44.5 (35)	46.5 (25)	0.886	41.5 (32)	44.0 (26)	0.347
QRS axis	48.0 (38)	49.0 (35)	0.845	51.0 (34)	49.0 (30)	0.545
T axis	29.5 (25)	28.0 (24)	0.572	30.0 (23)	28.0 (28)	0.691
Maximum P wave duration, ms	110.0 (12)	110.0 (12)	0.453	102.0 (12)	106.0 (12)	**0.002**
Minimum P wave duration, ms	82.0 (10)	84.0 (10)	0.073	76.0 (12)	84.0 (8)	**<0.001**
Mean P wave duration, ms	99.0 (8)	99.0 (9)	0.251	91.0 (10)	95.0 (7)	**<0.001**
P wave dispersion, ms	28.0 (12)	25.0 (12)	**0.039**	24.0 (12)	22.0 (12)	**0.004**
Maximum P wave amplitude, mV	0.12 (0.06)	0.12 (0.04)	0.883	0.12 (0.04)	0.12 (0.04)	0.783
Minimum P wave amplitude, mV	0.03 (0.01)	0.03 (0.02)	0.937	0.04 (0.02)	0.04 (0.01)	0.618
Mean P wave amplitude, mV	0.07 (0.02)	0.07 (0.02)	0.617	0.07 (0.03)	0.07 (0.02)	0.742
P wave amplitude- lead II, mV	0.11 (0.05)	0.11 (0.05)	0.407	0.11 (0.05)	0.11 (0.05)	0.998
P wave amplitude- lead V1, mV	0.08 (0.04)	0.07 (0.03)	0.370	0.07 (0.04)	0.07 (0.03)	0.883
P wave terminal force, mm•s	0.031 (0.04)	0.021 (0.03)	**0.023**	0.036 (0.04)	0.024 (0.04)	**0.030**

Values displayed are median (interquartile range).

Abbreviation: mm•s, product of millimetres and seconds.

P wave dispersion and PWTF-V_1_ were significantly lower in South Asians compared with White Europeans. South Asian females had increased P wave duration but no difference was seen in South Asian males. P wave amplitude was similar in both ethnic groups.

### Exercise test

Total exercise time was lower in South Asians and they achieved a lower maximum METs (Table 3). There was no difference in heart rate recovery at one minute.

**Table 3:** 24 hour electrocardiogram and heart rate variability data.

	Male			Female		
	Caucasian	South Asian	p-value	Caucasian	South Asian	p-value
Subjects	40	40		40	34	
Age, years	30.0 (6)	32.0 (11)	0.388	24.5 (11)	24.0 (9)	0.815
Length of recording, minutes	1332.8±88.5	1327.9±91.6	0.844	1350.7±78.9	1292.1±84.5	**0.015**
Heart rate (minimum)	49.0±6.1	53.8±6.7	**0.001**	53.6±7.7	56.2±4.8	**0.048**
Heart rate (maximum)	140.1±22.0	137.6±18.0	0.966	146.2±18.2	148.7±18.0	0.517
Heart rate (mean)	72.6±7.6	78.4±7.4	**0.001**	75.8±8.5	81.2±7.4	**0.003**
SVE total % of recording	0.0041 (0.004)	0.0015 (0.002)	**0.024**	0.0020 (0.004)	0.0000 (0.001)	**0.008**
*HRV*: *Time-* *domain measures*						
SDNN, ms	170.1 (59.6)	155.6 (36.6)	**0.011**	158.5 (61.4)	150.8 (44.3)	0.206
SDANN, ms	161.0 (54.9)	140.1 (46.2)	**0.036**	144.9 (47.6)	128.7 (36.4)	0.155
SDNN index, ms	68.8 (19.6)	57.0 (17.6)	**0.002**	63.5 (24.9)	57.8 (12.0)	0.326
RMSSD, ms	49.9 (26.3)	40.1 (16.8)	**0.013**	45.9 (29.9)	42.4 (20.9)	0.622
Triangular index	48.4 (18.2)	42.3 (11.0)	**0.010**	44.1 (23.4)	43.9 (12.2)	0.416
*HRV*: *Frequency-domain measures*						
VLF, ms^2^	1485 (684)	1184 (948)	0.065	1127 (869)	1048 (635)	0.812
LF, ms^2^	2004 (1212)	1274 (1423)	**0.005**	1341 (1091)	1285 (739)	0.803
HF, ms^2^	873 (1189)	780 (591)	0.079	941 (1189)	870 (1061)	0.610
LF/HF, %	3.4 (2.7)	3.1 (2.2)	0.909	2.5 (1.7)	2.3 (1.5)	0.789

Values displayed are mean ± SD or median (interquartile range).

Abbreviation: SVE, supraventricular ectopic; HRV, heart rate variability.

### 24 hour ECG and heart rate variability

South Asians had higher average heart rates and a significantly smaller burden of SVE (Table 4). South Asian males had reduced HRV in all time-domain measures and LF with a trend towards a lower VLF and HF (Table 4). No differences in HRV were seen in females.

**Table 4:** Exercise test results.

	Male			Female		
	Caucasian	South Asian	p-value	Caucasian	South Asian	p-value
Subjects	25	25		25	25	
Age, years	29.5±6.1	32.7±6.1	0.059	26.2±6.9	25.9±6.0	0.804
Resting heart rate, bpm	83.3±10.8	85.7±12.8	0.457	90.6±7.8	91.6±10.8	0.711
Resting systolic BP, mmHg	119.9±7.0	119.2±13.5	0.840	112.8±11.0	104.5±10.0	**0.005**
Resting diastolic BP, mmHg	71.5±6.2	77.4±8.2	**0.001**	72.2±10.3	66.1±8.0	**0.038**
Total exercise time, seconds	961.0±180.7	803.2±167.5	**0.002**	772.0±147.1	633.0±115.6	**<0.001**
Maximum heart rate, bpm	191.2±11.0	193.3±10.8	0.473	193.7±9.7	190.0±14.3	0.280
Maximum systolic BP, mmHg	159.2±14.6	157.8±15.0	0.722	137.7±13.9	138.3±16.1	0.883
Maximum diastolic BP, mmHg	70.0±7.6	70.7±6.7	0.794	69.9±8.9	70.5±16.4	0.209
% of target heart rate	100.0±5.3	103.3±5.1	**0.025**	100.1±4.6	97.7±6.5	0.140
Maximum METS achieved	18.7±3.3	15.7±3.1	**0.001**	15.2±2.9	12.5±2.1	**<0.001**
Heart rate at 1 minute recovery, bpm	160.7±13.4	166.2±12.1	0.122	163.3±11.1	160.7±19.3	0.565
Heart rate at 6 minutes recovery, bpm	108.3±12.4	117.6±9.9	**0.004**	108.3±9.5	111.0±16.8	0.479
Heart rate recovery- 1 minute, bpm	30.0±7.4	26.9±6.9	0.139	29.5±7.5	29.2±9.9	0.626

Values displayed are mean ± SD.

Abbreviations: bpm, beats per minute; BP, blood pressure; METS, metabolic equivalents.

Data from six South Asian females was invalid due to either artefact or prolonged removal of the ECG leads.

## Discussion

**Fig 1:**
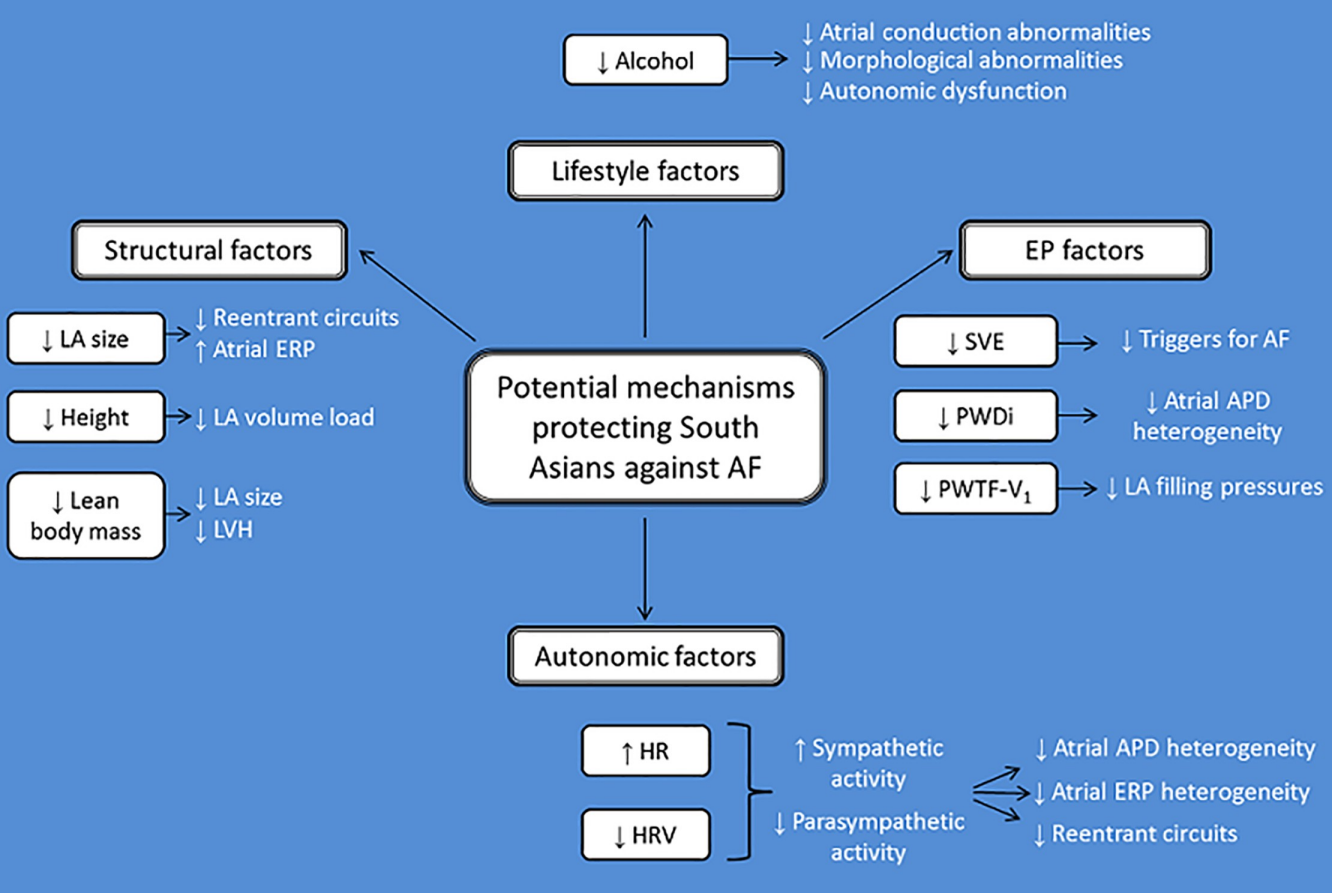
Summary of findings and their association with risk of atrial fibrillation. Abbreviations: LA, left atrial; ERP, effective refractory period; LVH, left ventricular hypertrophy; EP, electrophysiological; SVE, supraventricular ectopy; AF, atrial fibrillation; PWDi, P wave dispersion; APD, action potential duration; PWTF-V_1_, P wave terminal force in lead V_1_; HR, heart rate; HRV, heart rate variability.

This study provides normal reference ranges for ECG intervals and HRV in a young South Asian population without evidence of cardiovascular disease or its risk factors. Additionally, it demonstrates differences in anthropometric measurements, P wave indices and HRV between South Asians and White Europeans (Fig 1).

### Baseline characteristics and anthropometric measurements

Height, weight and BSA were all lower in South Asians, consistent with previous small observational studies[24,25]. Body size correlates with heart chamber dimensions and increasing height is closely related to increasing left atrial volume[26]. It is not surprising therefore that South Asians had a smaller left atrium compared with White Europeans although interestingly, left atrial size remained significantly smaller even after matching for BSA, suggesting that South Asians have proportionally smaller atria. Atrial enlargement is associated with a higher risk of AF[27] due to its effects on atrial refractory periods and the development of re-entrant circuits[28]. One study has also found that the impact of height on the risk of AF is independent of atrial size and it has been suggested that since increasing body size is related to increased stroke volume and cardiac output[29], the increased risk of AF could be linked to a higher volume load. Consequently, the reduced prevalence of AF seen in South Asians could relate to their smaller stature and reduced left atrial size.

Our findings show that South Asians have a significantly lower lean body mass, a measure which has recently been shown to be the predominant anthropometric risk factor associated with the occurrence of AF[30]. It has been speculated that high lean body mass increases stroke volume and cardiac output[31] which in turn can lead to the development of left ventricular hypertrophy[31] and atrial enlargement[31]. These structural changes provide favourable substrate for AF development. Whether lower lean body mass influences the risk of developing AF in South Asians remains to be determined.

Alcohol consumption was significantly lower in South Asians. Alcohol intake is closely associated with the development of AF, even when consumed at moderate levels[32] and this is believed to be due to its effects on atrial conduction, morphological alteration and autonomic impairment. Therefore, the lower level of alcohol consumption in South Asians may be protective against the development of AF.

### P wave indices

South Asians have significantly lower P wave dispersion and PWTF-V_1_ in comparison to White Europeans. P wave dispersion is a marker of inhomogeneity in the propagation of sinus impulses across the atria and is influenced by increases in atrial pressure, atrial size and metabolic stress. It has been shown to predict paroxysmal lone AF[17] as well as AF recurrence[33]. PWTF-V_1_ correlates with left heart filling pressures, is a specific indicator of left atrial enlargement and is associated with an increased risk of AF[18]. Reduced P wave dispersion and PWTF-V_1_ is consistent with South Asians having smaller atrial size. Variations in these indices may also reflect differences in the atrial conduction homogeneity and left heart filling pressures, both of which would influence AF risk, although more invasive techniques are required to confirm this.

South Asian females unexpectedly had a longer P wave duration compared with Caucasian females, implying differences in atrial conduction time. Studies have shown that both a prolonged[15] and shortened[15] P wave duration are associated with the development of AF. Therefore, the relevance of this finding in relation to the risk of AF in South Asians is uncertain.

### 24 hour ECG and heart rate variability

South Asians had a lower SVE burden in comparison with White Europeans although as one might expect in healthy cohorts, the overall burden of SVE was low in both groups. SVE detected on ambulatory monitoring has been shown to independently predict AF[34].

South Asians had a significantly higher minimum and mean heart rate compared with White Europeans. Heart rate is heavily influenced by the autonomic nervous system and a lower heart rate relates to a relative predominance of parasympathetic activity which in turn has been linked with a higher AF risk[35,36].

HRV was within normal range in all subjects. Interestingly, South Asian males had significantly lower SDNN, SDANN and triangular index in comparison with Caucasian males. These indices are all markers of overall HRV and mainly reflect increased sympathetic activity[37]. They also had lower RMSSD and a trend towards lower VLF and HF, all of which are markers of lower parasympathetic activity. Differences in HRV have previously been seen in South Asian children[38] but to our knowledge, this is the first time a difference has been demonstrated amongst South Asian adult males.

Overall, the differences seen in heart rate and HRV may reflect differences in autonomic function and would be most consistent with South Asians having sympathetic predominance. Whether this influences South Asians risk of developing AF is less clear.

### Exercise test

Total exercise time and METs achieved were significantly lower in South Asians implying that they are generally less physically active than White Europeans. There was a trend towards lower heart rate recovery in South Asian males, suggestive of higher sympathetic tone, but no difference was seen in females. The association between exercise and the risk of AF follows a J-shaped curve with increasing physical activity modestly reducing the risk of AF but extreme exercise, such as that performed by endurance athletes, increasing the risk of AF significantly. There are several different mechanisms to explain this. Exercise generally has a positive influence on risk factors such as hypertension, diabetes mellitus and obesity and has been shown to slow the age-related decline in arterial elasticity and associated cardiovascular disease. However, at high levels, exercise can lead to morphological changes such as left atrial enlargement and LVH and increases in vagal tone, all of which favour AF. Although lower exercise levels are likely to increase the risk of AF in South Asians, it is feasible that reduced activity might have the effect of minimising structural and autonomic changes within the heart.

### Limitations

This study should be interpreted in the context of certain limitations. Firstly, our findings are derived from non-invasive investigations and are consequently indirect measurements of atrial conduction and autonomic function. Nevertheless, we have identified significant differences between South Asians and White Europeans and our findings have highlighted areas of research for future clinical, genetic and experimental studies. Secondly, our reported reference ranges relate to healthy individuals aged 18 to 40 years of age and so may not be applicable to older subjects. Thirdly, these results are taken from a single-centre cohort and this may limit the generalisability of the findings.

## Conclusion

In comparison with White Europeans, South Asians are of a smaller stature with reduced lean body mass and smaller left atrial size. They have reduced P wave dispersion, indicative of less inhomogeneity of atrial conduction, and lower PWTF-V_1_, implying smaller atrial size and potentially lower left heart filling pressures. They also have a lower burden of SVE. Additionally, South Asians have increased mean heart rate and South Asian males have reduced heart rate variability, suggestive of sympathetic predominance. These morphological, electrophysiological and autonomic variations may help to explain why South Asians have a lower prevalence of arrhythmias such as AF.

## Supporting information

S1 File EDiSACA ECG data. (XLSX)

S2 File EDiSACA HRV data. (XLSX)

S3 File EDiSACA exercise test. (XLSX)

S4 File STROBE checklist (PlosONE). (DOC)
